# Automated Reinforcement Management System: Feasibility study findings of an app-based contingency management treatment for alcohol use disorder

**DOI:** 10.1016/j.dadr.2023.100140

**Published:** 2023-02-01

**Authors:** André Q. Miguel, Crystal L. Smith, Nicole M. Rodin, Ron K. Johnson, Michael G. McDonell, Sterling M. McPherson

**Affiliations:** a Elson S Floyd College of Medicine, Washington State University, Spokane, WA, United States; b College of Pharmacy and Pharmaceutical Sciences, Washington State University, Spokane, WA, United States; c Managed Health Connections, Spokane, WA, United States; d Analytics and PsychoPharmacology Laboratory, Spokane, WA, United States; e Program of Excellence in Addictions Research, Spokane, WA, United States

**Keywords:** Alcohol use disorder, Contingency management, Digital therapeutics, Telehealth, remote treatment

## Abstract

Alcohol Use Disorder (AUD) is the most prevalent substance use disorder in the United States and is directly related to 5% of all annually reported deaths worldwide. Contingency Management (CM) is among the most effective interventions for AUD, with recent technological advancements allowing CM to be provided remotely.

**Objective:** To evaluate the feasibility and acceptability of a mobile Automated Reinforcement Management System (ARMS) designed to provide CM for AUD remotely.

**Methods:** Twelve participants with mild or moderate AUD were exposed to ARMS in a A-B-A within-subject experimental design where they were required to submit three breathalyzer samples per day. During the B phase participants could earned rewards with monetary value for submitting negative samples. Feasibility was determined by the proportion of samples submitted and retention in the study and acceptability was based on participants self-reported experience.

**Results:** The mean number of samples submitted per day was 2.02 out of 3. The proportion of samples submitted in each phase was 81.5%, 69.4% and 49.4%, respectively. Participants were retained for a mean of 7.5 (SD=1.1) out of 8 weeks with 10 participants (83.3%) completing the study. All participants found the app easy to use and stated it helped them reduce their alcohol use. Eleven (91.7%) would recommend the app as an adjunct to AUD treatment. Preliminary indicators of efficacy are also presented.

**Conclusions:** ARMS has shown to be feasible and well accepted. If shown effective, ARMS can serve as an adjunctive treatment for AUD.

## Highlights

• ARMS enables CM to be provided remotely.
• ARMS is feasible and well accepted by participants.
• ARMS holds promise as an adjunctive treatment for AUD.

## 1 Introduction

With a lifetime and past-year prevalence of 29.1% and 13.9%, respectively, Alcohol Use Disorder (AUD) is the most prevalent substance use disorder in the United States (Degenhardt et al., 2018; Grant et al., 2004). Worldwide, alcohol use is directly related to over 5% of all annually reported deaths, being a causal factor for more than 200 diseases, injuries, and conditions (WHO, 2018). Contingency Management (CM) is a behavioral intervention for substance use disorders that provides reinforcers (i.e., rewards with monetary value) contingent on objective verification of drug abstinence through the submission of negative toxicological specimens (Higgins et al., 1991).

Recent technological advances have enabled the development of mobile applications (apps) and low-cost breathalyzer devices that can be purchased by consumers (You et al., 2017). Together, these technologies allow for the remote monitoring of alcohol use and provision of inexpensive and accessible CM for AUD. Studies that have combined mobile apps and remote breathalyzers to provide CM have found it to be an effective strategy to reduce alcohol use and promote alcohol abstinence (Alessi and Petry, 2013; Koffarnus et al., 2018; Oluwoye et al., 2019). Despite these promising results however, app-based CM for AUDs has yet to be integrated into real-world treatment practices and evaluated in community treatment settings. Furthermore, to our knowledge, there is currently no available software to provide CM in a completely automated manner, including objective identity verification.

We developed an integrated, completely automated, end-to-end CM system intended to enable community treatment programs to deliver CM remotely. The objective of this study was to evaluate the feasibility, acceptability, and initial efficacy of the mobile Automated Reinforcement Management System (ARMS) to provide CM for AUD remotely.

## 2 Material and methods

### 2.1 Automated Reinforcement Management System (ARMS)

ARMS is a HIPAA compliant hybrid interface that includes a mobile-app for patients and a web-based platform for treatment providers. ARMS was developed to allow clinicians to provide adequate CM treatment for AUD remotely to anyone who owns a smartphone. Notably, ARMS includes other important features that can be critical during the management of AUD. For instance, ARMS collects Ecological Momentary Assessments (EMA) created based on the Addiction Neuroclinical Assessment (ANA) framework (Kwako et al., 2017; Kwako et al., 2016) and GPS location “geofencing”. ARMS uses this information, together with the Breath Alcohol Content (BrAC) results, and applies machine learning to create an “eminent relapse risk score”. Features also include the ability to inform the patient, provider or an “important other” of the risk of eminent relapse based on this risk score. A full description of ARMS's capabilities as well as data on provider feedback considered in its development have been previously published (Miguel et al., 2021; Smith et al., 2022).

### 2.2 Delivering remote contingency management using ARMS

**Fig. 1:**
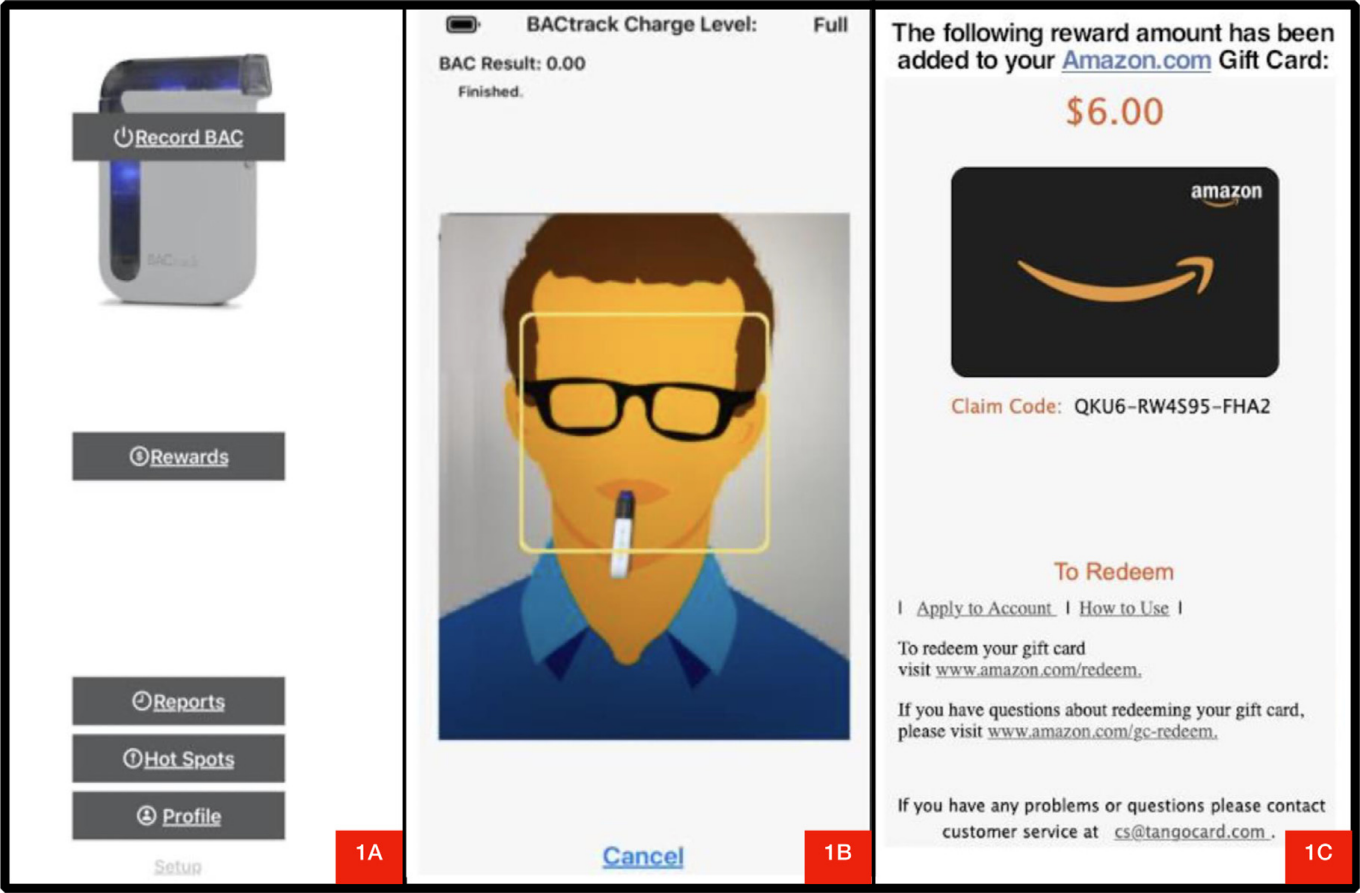
Patient-facing screenshots from the Automated Reinforcement Management System mobile app.

In ARMS, the app syncs via Bluetooth with commercially available electronic breathalyzer devices (e.g., BACtrack), enabling the submission of BrAC samples through the app. At pre-determined times of the day, the app cues patients via push messages to submit a breathalyzer sample. After opening the app and clicking on “submit sample” box (Fig. 1a), ARMS instructs patients to place their face in the center of a circle displayed on their phone screen while blowing into the breathalyzer (Fig. 1b). A photo is taken using the mobile camera while participants are blowing into the breathalyzer, which allows for facial recognition of the person submitting the sample. ARMS uses Amazon Rekognition (AR) technology to compare sample submissions to a reference image captured at baseline. Thus, enabling ARMS to validate participants identity automatically and instantly. However, given the need to evaluate the sensitivity and specificity of this feature, research staff validated the identity for each submission. This was done by comparing images taken during the study with an image of the same person taken during account setup at baseline.

**Fig. 2:**
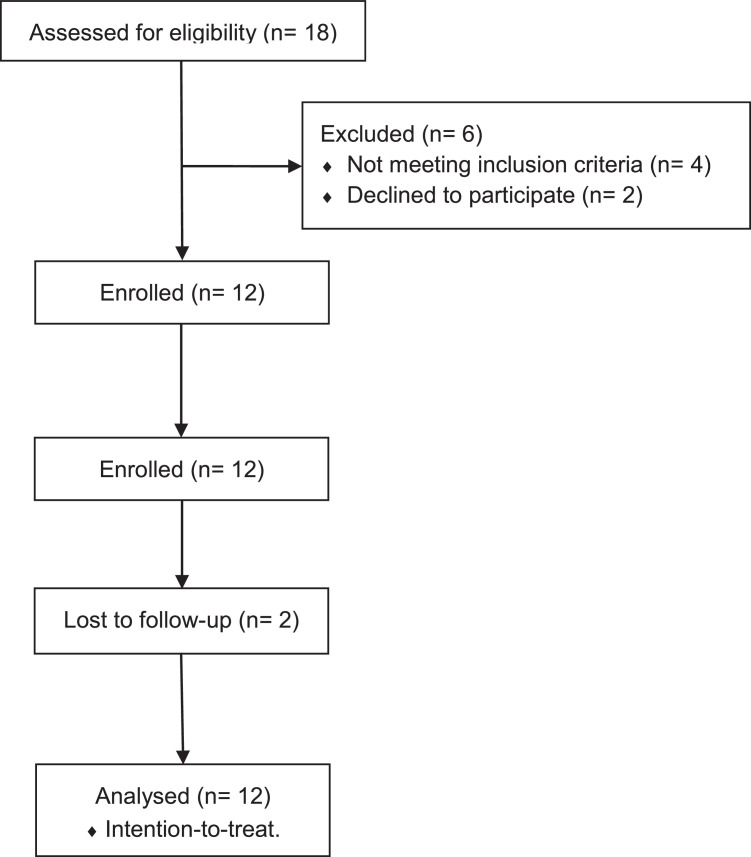
CONSORT flow diagram.

After submission, participants received a message that the sample was received and was awaiting verification. A research coordinator had 24 hours to confirm (or not) the identity. If the image and alcohol abstinence was confirmed (i.e., BrAC=0.00), participants were immediately informed of the amount of rewards earned (Fig. 1c) and allowed to choose to bank their earnings for future withdrawal or use the app to immediately redeem their rewards. Participants could choose to redeem any specific amount in the form of a digital gift card (e.g., amazon, tango) sent to participants’ preregister email address immediately after request (Fig. 2).

### 2.3 Study design

We used an A-B-A within-subject experimental design. The two A phases lasted for two weeks each and the B phase lasted four weeks. During both A and B phases, participants were asked to use the app to submit three breathalyzer samples per day (9am, 2pm and 8pm). Participants were prompted by the app to record and submit their samples within a half-hour window of these pre-determined times. During the A phases (control conditions), participants earned $2.00 for every sample submitted within the pre-determined window, independent of sample results. During the B phase (CM condition), participants received $2.00 for the first alcohol-negative breath sample submitted (BrAC=0.00), with this amount escalating by $0.25 per alcohol negative sample submission consecutively to a maximum value of $3.50. Vouchers were reset to $2.00 if the participants failed to submit a sample, submitted an alcohol-positive sample (BrAC>0.00), or could not be identified in the image captured. This escalating reinforcement schedule with the reset component after a positive or missing sample submission is among the most commonly used CM schedules (Burduli et al., 2018; Garcia-Rodriguez et al., 2009; Miguel et al., 2022) after it was shown to be more effective in promoting continuous abstinence and preventing relapse when compared to other CM reinforcement schedules (Roll et al., 1996). Participants could earn up to $456.75 in rewards if all BrAC samples were submitted at the pre-defined times and tested negative for alcohol. Notably, this first iteration of ARMS was developed for IOS systems only, as such, if a participant did not own an iPhone (8 participants total), one was provided to them for the duration of the study.

### 2.4 Participants

To be eligible, participants needed to be 18 or older, have an Alcohol Use Disorders Identification Test (AUDIT) (Saunders et al., 1993) score of eight or higher (i.e., according to the World Health Organization guidelines, scores of eight or higher suggest hazardous or harmful drinking) (Babor et al., 2001) and be interested in reducing their drinking. In addition, participants also needed to be able to read and speak English, provide written informed consent, and live in the Spokane, WA and Coeur d'Alene, ID area. Participants with severe AUD or a psychotic disorder according to the Diagnostic and Statistical Manual of Mental Disorders, 5^th^ edition (DSM-5) (APA, 2013), with significant risk of dangerous alcohol withdrawal, with a lifetime suicide attempt, or suicidal ideation in the past year were not eligible to enroll in the study.

### 2.5 Feasibility, acceptability, and efficacy measures

Feasibility was determined by the proportion of samples submitted and study retention (i.e., days elapsed since study enrolment and last sample submitted using the ARMS app). Acceptability was evaluated using a semi-structured questionnaire on participants’ experience with the app, collected at the end of the study. Sensitivity and specificity analyses of AR were conducted considering research staff's facial image validation as the gold standard and an 80% match on AR as the cut-off. Efficacy was determined by the proportion of alcohol-negative breath samples submitted during each study phase.

### 2.6 Statistics

To determine the effect of the CM app on the proportion of alcohol-negative samples submitted, we used generalized estimating equations (GEE) where the proportion of alcohol-negative samples submitted was the primary outcome, phase (A-B-A) was the primary predictor of interest, and baseline Ethylglucuronide (EtG) result, age and sex were included as covariates. Phase B (CM condition) was set as the reference group. Analyses were conducted using STATA 15 with the alpha level set at 0.05.

## 3 Results

### 3.1 Demographics

This study included a total of n=12 participants. The sample was predominantly male (n=9; 75%), with a mean age of 39.5 (SD=8.6) years, and entirely White/Caucasian. A fourth of the sample was unemployed (n=3) and 91.7% (n=11) tested positive for EtG (500 ng/mL) at baseline, indicative some level of heavy drinking in the last three to four days (McDonell et al., 2015).

### 3.2 Feasibility indicators

The total number of samples submitted was 1360 out of 2016 (69.9%) possible submissions, with a mean of 2.02 samples submitted per day (SD=1.2). Of these, 189 samples (13.9%) tested alcohol-positive and varied from BrAC=0.01-0.21. In terms of number of BrAC samples submitted by phase, a total of 411 samples were submitted during the first A phase (81.5%), 700 samples were submitted during the B phase (69.4%), and 249 samples were submitted during the second A phase (49.4%). In addition, of the 12 participants enrolled in the study, 10 of them (83.3%) submitted at least 55% of the total possible samples (i.e., 748 or more samples submitted) and seven of them (58.3%) submitted at least 75% of the total possible samples (i.e., 1020 or more samples submitted). However, six participants (50%) reported problems with the app at the time of the sample submission (e.g., login error) which did not allow them to submit a sample. Participants were retained for a mean of 7.5 weeks (SD=1.1) with 10 participants (83.3%) submitting BrAC samples until the last day of this 8 week experiment.

### 3.3 Acceptability indicators

All participants (100%) found the app easy to use and stated that it helped them reduce their alcohol use. Eleven (91.7%) would recommend the app as an adjunct to AUD treatment. Participants also reported frustrations with the app and suggestions for improvement. Seven participants (58.3%) suggested that increasing the 30-minute window to one hour would enable users to submit the sample on time and four participants (33.3%) suggested developing the app for android systems. Six participants (50%) reported being very frustrated with the app due to problems experienced that did not allow them to submit their samples (one participant dropped out for that reason).

### 3.4 Amazon recognition performance

In order to evaluate the sensitivity and specificity of AR, a cut-off of 80% match was selected to to determine participants’ identity. As a result, of the 1,360 images submitted; AR found that 1,266 achieved at least an 80% match (93.1%). Of the 94 images with less than 80% match, research staff were only unable to confirm participants’ identity in 13 of them (i.e., true negatives; 13.8%), yielding an AR sensitivity of 94%. Each time AR was unable to confirm a participants’ identity, images were of low quality and included one or more of the following characteristics: blurred images, dark images, and none face-centered images.

Regarding specificity, research staff was unable to confirm participants’ identity in only 13 images (i.e., true negative). All of which were considered not a match by AR, indicating a 100% specificity rate. However, due to the small number of true negative images in this data, further studies are needed for a better understanding of the specificity of the AR technology when applied for this context.

### 3.5 Proportion of negative samples submitted

As can be seen in Table 1, the proportion of alcohol-negative BrAC samples submitted by phase was 68.8%, 61.1% and 41.5%, respectively. The proportion of alcohol-negative BrAC samples submitted during the B phase (CM condition) was significantly higher than the second A phase (control condition) (B=-17.84; *p*=0.03). Holding constant the effect of the covariates (age, sex, baseline EtG), the proportion of alcohol-negative BrAC samples submitted was 17% smaller during the second A phase when compared to the B phase. No other statistically significant differences were observed.

**Table 1:** Proportion of BrAC negative samples submitted by treatment phase.

	n° of negative BrAC submitted	% of negative BrAC submitted	B	95% CI	*p*-value
Phase B* (experimental)	615	68.8%	-	-	-
Phase A, first (control)	347	61.1%	7.41	-8.81 – 23.44	0.370
Phase A, second (control)	209	41.5%	**-17.84**	**-34.07 – -1.61**	**0.031**
Sex	-	-	-23.1	-61.57 – 15.38	0.239
Age	-	-	0.59	-1.07 – 2.25	0.484
Baseline Etg	-	-	14.15	-24.03 – 52.34	0.468

⁎ = reference group; BrAC = Brearh Alcohol Content; n° = number; % = proportion; Etg = Ethylglucuronide; B = unstandardized beta; CI = Confidence Interval; bolded rows indicate significance.

## 4 Discussion

To our knowledge, ARMS is the first app designed to provide a completely automated CM intervention for AUD. The high rate of samples submitted (2/3 per day), the high retention rate (83.3% of participants completed the study), and the positive feedback indicates that this app is both feasible and acceptable. Furthermore, the high sensitivity and specificity indicates that AR enables completely automated system of CM with objective identification verification. The incorporation of this feature should have a direct effect on costs related to labor as well as may improve the effectiveness of remotely delivered CM technologies by making rewards available immediately after a negative BrAC sample is submitted (i.e., the closer rewards are made available after a specific response is emitted, the stronger it the reinforcing effect that reward has over that response) (Odum, 2011).

The higher proportion of alcohol-negative samples submitted during the CM condition compared to second the control condition offers a signal of efficacy as well. Such findings are aligned with two pilot studies, with similar sample sizes, designed to evaluate the remote delivery of CM for AUD treatment (Koffarnus et al., 2018; Oluwoye et al., 2019). It is important to not however, that no significant difference was observed on alcohol-negative samples submitted when comparing the CM condition with the first control condition. As such, it is possible that changes on pattern of use or interaction with the app over the course of the study may have contributed to the observed results. Thus, the conduction of a randomized controlled trial, is still necessary to be able to determine the potential efficacy of ARMS.

### 4.1 ARMS 2.0

The next iteration of ARMS will include several necessary changes. First, we will use AR to validate participants identity. This will allow ARMS to deliver reinforcers in a completely automated manner immediately after a sample is submitted. In addition, every time a participants’ identity cannot be confirmed via AR, participants will be informed immediately that their identity was not confirmed and will be offered the opportunity to submit another sample. Second, following participant feedback, the time window for sample submission will increase from 30 minutes to one hour. Third, as mentioned above, a substantial number of participants (50%) reported not being able to submit a sample at the predetermined time due to a login error. Enhancement of the transmission algorithm for reportback will be tested in future versions for low bandwidth environments as well as alternative approaches that enable the secure storage and forward submissions of samples collected in the absence of internet connection. Lastly, we will also develop an android version of ARMS allowing anyone owning a smartphone to have access to this technology.

### 4.2 Limitations

This study has some limitations that should be noted. First, our small sample size may have reduced our ability to uncover differences due to our low statistical power. Second, our sample was composed entirely by white individuals limiting our ability to generalize our findings to other race/ethnicities. Third, its well documented that the efficacy of CM decreases when access to the reinforcers (e.g., rewards) are delayed (Critchfield and Kollins, 2001; Madden et al., 1997). In this study in order to get access to the electronic gift card consequent to the submission of a negative BAC sample, the picture captured at the time of the sample submission need to be validated. This process was done manually and as a result, participants could have to wait up to 24 hours to get access to the electronic gift card. Thus, it is possible that delayed access to the reinforcer may have reduced the efficacy of this intervention. Fourth, the last BAC submission was set at 8pm, as such it is possible that participants may have consumed alcohol after submitting the last daily sample. Alternative schedules for submitting samples should be explored in order to overcome this limitation. Lastly, only individuals with a mild or moderate AUD were included in this study where no form of usual treatment was provided. As such, it is unclear how ARMS would perform in terms of feasibility and acceptability in a population with a more severe AUD currently enrolled in community- based AUD treatment.

## 5 Conclusion

Despite technological issues that need be addressed for the next phase, ARMS has shown to be feasible and acceptable. If shown effective, ARMS can serve as an ultra-low barrier adjunctive treatment for AUD that can be particularly useful to rural -based community treatment programs.

## Contributors

AQM, CLS, RKJ, MGM, NMR, and SMM contributed to the design of this study. AQM, CLS, and NMR collect data. AQM and CLS analyzed and interpreted the data with the oversight of SMM. AQM wrote the first draft of the paper. CLS, NMR, RKJ, MGM, and SMM contributed to the writing and/or revision of the manuscript. All authors read and approved the final manuscript.

## Declaration of Competing Interest

None.
